# A combined model of BCVA, TRAb, and NLR predicts response to intravenous methylprednisolone in dysthyroid optic neuropathy

**DOI:** 10.3389/fmed.2026.1811285

**Published:** 2026-05-04

**Authors:** Shu Liu, Linfeng He, Ruili Wei

**Affiliations:** Department of Ophthalmology, Shanghai Changzheng Hospital, Second Affiliated Hospital of Naval Medical University, Shanghai, China

**Keywords:** dysthyroid optic neuropathy, graves orbitopathy, intravenous methylprednisolone, neutrophil-to-lymphocyte ratio, thyrotrophin receptor antibody

## Abstract

**Background:**

High-dose intravenous methylprednisolone (HD-IVMP) is the first-line treatment for dysthyroid optic neuropathy (DON). However, a significant proportion of patients exhibit a poor response, risking irreversible vision loss when surgical decompression is delayed. Identifying reliable predictors for failure of HD-IVMP is crucial for optimizing treatment strategies. This retrospective study aimed to identify risk factors associated with a poor response to HD-IVMP in DON patients, develop a predictive model for clinical decision-making, and assist in determining the optimal timing of orbital decompression surgery.

**Methods:**

A retrospective cohort study was conducted involving 44 patients (44 eyes) diagnosed with DON and treated with HD-IVMP. Basic characteristics, clinical features, orbital computed tomography (CT) findings, and laboratory parameters were analyzed. Treatment response was defined as complete visual recovery without requiring surgical decompression within 6 months. Least absolute shrinkage and selection operator (LASSO) and multivariable logistic regression analyses were used to screen and identify independent predictive factors. The predictive performance of the model was evaluated by plotting the receiver operating characteristic (ROC) curve. Additionally, decision curve analysis (DCA), net reclassification index (NRI), integrated discrimination improvement (IDI), and bootstrap internal validation were used to evaluate the clinical utility of the model.

**Results:**

At the 6-month follow-up, 20 of the 44 (42.5%) eyes achieved complete visual recovery and consequently avoided surgical decompression. A multivariable analysis identified three independent predictors of non-response to HD-IVMP: poorer baseline best-corrected visual acuity (BCVA; odds ratio [OR] = 1.43, 95% CI: 1.05–1.95, *p* = 0.04), higher thyroid-stimulating hormone receptor antibody (TRAb) levels (95% CI: 1.02–2.61, p = 0.04), and elevated NLR (OR = 1.30, 95% CI: 1.04–1.63, *p* = 0.02). A combined predictive model integrating these factors demonstrated superior performance (AUC = 0.944) with a sensitivity of 88% and a specificity of 85%, exceeding any single predictor. The internal validation also confirmed the model’s robust clinical utility.

**Conclusion:**

This study establishes that baseline BCVA, TRAb, and NLR are significant and readily available risk factors for failure of HD-IVMP in DON. The developed combination model provides clinicians with a valuable tool for the early identification of patients who are unlikely to benefit from medical therapy alone, thereby facilitating timely surgical intervention to prevent permanent visual damage and improve overall management outcomes. External validation of this model is required in future prospective studies.

## 1 Introduction

Graves’ orbitopathy (GO), the principal extrathyroidal manifestation of Graves’ disease (GD) (1), is an autoimmune disease characterized by inflammation that features the expansion of orbital tissue volume, including extraocular muscles, orbital connective tissue, and adipose tissue, followed by eyelid retraction, edema, proptosis, restricted eye movement, and diplopia (2). Acute dysthyroid optic neuropathy (DON) is the most severe vision-threatening complication of GO, resulting from mechanical compression and ischemic injury to the optic nerve. Currently, there are numerous diagnostic criteria available for assessing DON, such as the European Group on Graves’ Orbitopathy (EUGOGO) (1) and the Bartley criteria (3). However, no unified consensus on the clinical diagnosis of DON has yet been established.

Meanwhile, the most challenging aspect of DON involves not only accurate diagnosis but also therapeutic strategies (4). With an incidence of 3–7% in patients with GO (5), DON necessitates urgent intervention to prevent irreversible vision impairment or even vision loss (6). The currently recommended first-line treatment is high-dose intravenous pulse methylprednisolone (HD-IVMP), based on the majority of existing research (5). Although approximately 40% of patients achieve significant visual recovery with HD-IVMP (7, 8), a substantial proportion of patients experience incomplete responses, highlighting a critical heterogeneity in treatment outcomes. Therefore, visual function and ocular signs should be closely monitored during HD-IVMP treatment. Once patients show a poor response to HD-IVMP, emergency orbital decompression surgery should be performed to preserve vision (9). Such surgery can rapidly increase orbital volume and reduce orbital pressure, thereby alleviating optic nerve compression.

Unfortunately, no explicit factors that correlate with the response to HD-IVMP treatment in DON have yet been identified. Thyroid-stimulating hormone receptor antibodies (TRAb) have been proposed as a potential marker for the HD-IVMP treatment of GO (10), but their specific role in DON remains unconfirmed. Furthermore, only a single-center analysis revealed that, in patients with DON, advanced age, prolonged disease duration, poor baseline visual acuity, and exophthalmos are key risk factors for a poor response to HD-IVMP treatment (11). However, this study included only clinical features, and approximately half of the patients did not undergo orbital computed tomography (CT) scans. This indicates that the predictive value of multifactorial characteristics has not been fully investigated in the absence of confirmed apical crowding.

Therefore, finding cost-effective, readily available, and commonly used indicators, such as laboratory biomarkers, is important for guiding treatment regimens in DON. This study aimed to identify clinical and laboratory factors that independently correlate with visual outcomes following HD-IVMP therapy for DON. By using LASSO and multivariable analysis, we identified disease-related predictors of response to HD-IVMP treatment and constructed a model to predict treatment response based on these factors. Our goal is to provide a tool to distinguish patients who are optimal candidates for medical therapy from those who may require early surgical intervention, thereby avoiding delays in the optimal timing for surgery.

## 2 Materials and methods

### 2.1 Patients

A total of 54 patients who met the diagnostic criteria for DON at the Department of Ophthalmology of the Second Affiliated Hospital of Naval Medical University were retrospectively reviewed between September 2018 and August 2025. The enrolled patients were first-onset cases with no prior treatment with IVMP. For cases affecting both eyes, the more severely affected side was included. The diagnosis of DON was established based on at least two clinical features indicative of optic neuropathy (12): a decrease of two or more lines in best-corrected visual acuity (BCVA), impaired color vision, optic disc edema, visual field defects, and a relative afferent pupillary defect (RAPD), without other explanation. Radiographic criteria, adapted from Giaconi et al. (13), required evidence of apical crowding on orbital CT, characterized by enlargement of the extraocular muscles, proliferation of orbital fat tissue, and/or a muscle index greater than 50%. There were three eyes that were exceptions, being clinically diagnosed with DON despite no evidence of apical crowding.

**Figure 1:**
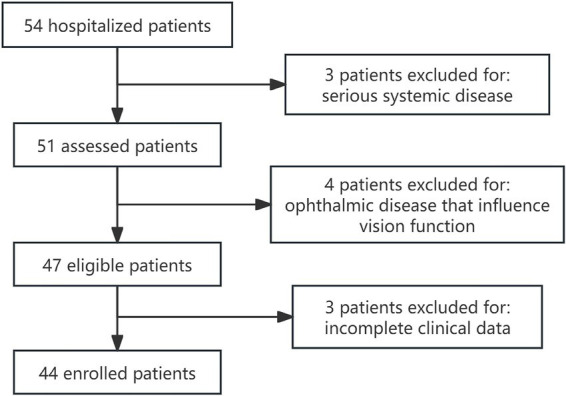
Flow diagram shows the patient selection process.

Among the 54 patients, 3 patients were excluded due to serious systemic diseases, such as poorly controlled hypertension, poorly controlled diabetes mellitus, active infectious diseases, and a prior history of orbital surgery. An additional 4 patients were excluded because of ophthalmologic diseases that could significantly impair visual function, such as cataracts, glaucoma, macular degeneration, or congenital, ischemic, or inflammatory optic neuritis. Additionally, 3 patients were excluded due to incomplete clinical data. As a result, the final study cohort comprised 44 patients, as illustrated in the flowchart in Figure 1. The study was approved by the ethics committee of our institution. All enrolled patients provided written informed consent, agreeing to the treatment protocol and permitting the use of their data for statistical analysis.

### 2.2 Treatment schedule and response criteria

All patients were treated with either 500 or 1,000 mg of HD-IVMP daily for 3 consecutive days, according to Jeon et al. (14). The treatment was repeated the next week, followed by tapering of steroids via oral or intravenous administration. For patients with persistent active inflammatory GO (clinical activity score, CAS ≥ 3) in at least one orbit, weekly HD-IVMP (250 mg or 500 mg) was continued until inactivation was achieved following medical decompression. However, the cumulative total dose throughout the entire treatment process was strictly limited to a maximum of 10 g. During the baseline period and throughout follow-up, blood glucose, liver function, heart rate, blood pressure, and any adverse reactions were closely monitored.

In this study, an improvement in BCVA of less than two lines after 2 weeks of medical decompression therapy was considered an inadequate visual response to steroids. Surgical decompression was then performed in these eyes, regardless of the degree of GO activity. Eyes that initially responded to steroids but subsequently experienced DON recurrence were also treated with surgical decompression. Visual function was considered fully recovered when at least two parameters were recorded—specifically, best-corrected visual acuity (BCVA) of ≤0.10 logMAR, less than three errors in the Hardy–Rand–Rittler pseudoisochromatic tables (HRR second edition, Richmond products, Albuquerque, NM) test, or visual field changes (mean deviation [MD] ≥ −3 dB or pattern standard deviation [PSD] <3 dB or both). Finally, eyes with recovered visual function and without the need for surgical intervention at 6 months after drug-induced decompression therapy were defined as treatment responsive (primary outcome) (7).

### 2.3 Ophthalmologic examination

In the study, all patients underwent a comprehensive ophthalmologic examination based on the EUGOGO criteria (1). BCVA [RT-5100, NIDEX] was assessed in all patients, and the results were recorded and expressed as the logarithm of the minimum angle of resolution (logMAR). Color vision was tested using Ishihara plates and considered abnormal if the patient misread two or more plates. All patients underwent visual field testing with Humphrey visual field analyzer [HFA740i, Carl Zeiss Meditec]. Visual field examinations were repeated for those with reliability indices outside the normal range (fixation loss rate of >20%, false positive and false negative rates of >33%). If the results remained unreliable after retesting, they were excluded from the analysis. The baseline visual field assessment criteria were as follows: a visual field was deemed abnormal if at least one global index (mean deviation [MD] or pattern standard deviation [PSD]) fell outside the normal range (*p* < 0.05). Abnormal visual fields were retested before treatment to confirm the diagnosis. In addition, each patient underwent a routine slit-lamp examination, RAPD test, intraocular pressure (IOP) (TX-20, Canon), fundus imaging (APS-70X, Kanghua), and optical coherence tomography (OCT) scans [Spectralis OCT, Heidelberg]. The optic disc swelling was assessed via a fundus examination using a + 90 diopter lens [V90C, Volk].

The CAS was used to assess the inflammatory activity of GO in each orbit. According to the standard criteria (15), a CAS of ≥4/10 was classified as active GO.

### 2.4 Imaging and laboratory assessments

All enrolled patients underwent orbital imaging examinations, which were uniformly performed using the same CT scanner (Philips Spectral 256) and evaluated by two dedicated radiologists. Signs of apical crowding were noted according to McKeag et al. (16), including increases in orbital tissue volume with enlargement of the extraocular muscles and/or orbital fat expansion.

Laboratory characteristics, tested from the patients’ serum, including thyroid-stimulating hormone (TSH, 0.27 ~ 4.2 mIU/L), free triiodothyronine (FT3, 3.1 ~ 6.8 mmol/L), free thyroxine (FT4, 12 ~ 22 mmol/L), triiodothyronine (T3, 1.3 ~ 3.1 mmol/L), thyroxine (T4, 66 ~ 181 mmol/L), TRAb (<1.75 IU/L), anti-thyroglobulin antibodies (TGAb, <115 IU/mL), and anti-thyroid peroxidase autoantibody (TPOAb, <34 IU/mL), were collected from electronic medical records. Additionally, the neutrophil-to-lymphocyte ratio (NLR) was calculated from the patients’ complete blood count results. A high NLR has been shown to predict poor outcomes in various inflammatory diseases, such as ankylosing spondylitis (17), diabetic microvascular complications (18), and chronic kidney disease (19). Additionally, studies have shown that NLR can be identified as a potential marker in the progression of active GO and relapse of GO after anti-thyroid drug therapy (20–22).

### 2.5 Statistical analysis

Continuous variables were expressed as descriptive statistics in the form of means ± standard deviations (SD) across groups, whereas categorical variables were presented as the number of events observed. The CAS was presented as the median with range. Comparisons of differences between the groups for categorical variables were performed using the chi-squared test; comparisons of thyroid function-related data were performed using the Mann–Whitney U-test; and for all continuous variables, independent samples t-tests were used to compare group means, assuming equal variances. The study employed LASSO and the multivariable logistic regression analysis to identify independent predictors of response of visual function recovery following medical decompression therapy. The results were presented as odds ratios with 95% confidence intervals. Differences were considered statistically significant when *p*-values were < 0.05. Receiver operating characteristic (ROC) curve analysis was performed to evaluate the diagnostic performance of each indicator. All statistical analyses were conducted using R software (version 4.5.2, R Foundation for Statistical Computing).

This study also employed decision curve analysis (DCA) using R software with the dcurves package. This method generates decision curves by plotting the functional relationship between net benefit and a series of threshold probabilities, thereby evaluating the potential clinical benefit of specific indicators or models. This approach aims to identify clinically significant threshold probability intervals while quantifying the benefit level that biomarkers or models can provide within these intervals. In addition, to investigate whether the predictive performance significantly improved as more parameters were incorporated into the model, the net reclassification index (NRI) and integrated discrimination improvement (IDI) were calculated using the PredictABEL package. Calibration curves and ROC analyses were also plotted via 1,000-round bootstrap resampling using R software to comprehensively evaluate the predictive performance of the model.

## 3 Results

### 3.1 DON characteristics at presentation and treatment

A total of 44 eyes (44 patients) fulfilled our inclusion criteria, and their data were used for further analyses. Among them, 15 patients had bilateral DON, in which the more severely affected side was included. All 44 DON patients received intravenous methylprednisolone 500 mg–1 g daily for 3 consecutive days according to the aforementioned protocol, repeated the following week, and then oral prednisolone therapy was continued. As shown in Table 1, the treatment responsive group (medical decompression only) comprised 20 eyes, and the unresponsive group (surgical decompression) comprised 24 eyes. The mean age of the participants was 50.80 ± 9.08 years, with no significant difference in the mean age between the groups (responsive outcome group: 49.05 ± 10.12 years vs. unresponsive outcome group: 52.25 ± 8.03 years, *p* = 0.73, Table 1). There was no statistically significant difference in sex distribution between the two groups (*p* = 0.80).

**Table 1:** Baseline clinical parameters of the eyes affected with dysthyroid optic neuropathy treated with high-dose intravenous methylprednisolone.

Variables	All eyes (*n* = 44)	Responsive eyes (*n* = 20)	Unresponsive eyes (*n* = 24)	*p*-value
Age (years)	50.80 ± 9.08 (21–71)	49.05 ± 10.12 (21–63)	52.25 ± 8.03 (38–71)	0.73^c^
Sex (Male/Female)	21/23	8/12	13/11	0.80^a^
Smoking (yes/no)	15/29	7/13	8/16	0.91^a^
Diabetic (yes/no)	2/42	1/19	1/23	0.30^a^
GD duration (months)	22.63 ± 29.34	27.68 ± 40.11	18.63 ± 16.57	0.32^c^
GO duration (months)	14.7 ± 14.83	11.50 ± 12.58	17.38 ± 16.25	0.19^c^
GO CAS (range)	4 (1–7)	4 (3–5)	5 (1–7)	0.05^c^
BCVA (logMAR)	0.57 ± 0.56	0.27 ± 0.26	0.82 ± 0.62	**<0.001** ^**c**^
Exophthalmos (mm)	21.47 ± 3.62	20.23 ± 3.26	22.5 ± 3.65	**0.04** ^**b**^
Optic disc swelling (yes/no)	17/27	2/18	15/9	**<0.001** ^**a**^
Apical crowding (yes/no)	41/3	18/2	23/1	0.58^a^
MD (decibel)	−10.72 ± 9.87	−5.56 ± 6.67	−15.02 ± 10.14	**<0.001** ^**c**^
PSD (decibel)	5.24 ± 3.20	4.07 ± 2.47	6.23 ± 3.45	**0.03** ^**c**^
VFI (decibel)	69.09 ± 33.02	84.45 ± 22.34	56.29 ± 35.35	**<0.01** ^**c**^
Color vision defect (yes/no)	34/10	15/5	19/5	0.51^a^
Thyroid laboratory results (normal range)				
T3 (nmol/L)	2.05 ± 0.59	1.94 ± 0.49	2.14 ± 0.65	0.26^b^
T4 (nmol/L)	98.77 ± 22.87	103.8 ± 21.27	94.56 ± 23.74	0.18^b^
Free T3 (pmol/L)	5.13 ± 1.14	4.91 ± 0.84	5.31 ± 1.33	0.25^b^
Free T4 (pmol/L)	15.91 ± 3.26	15.97 ± 2.96	15.86 ± 3.55	0.91^b^
TSH (mIU/L)	2.00 ± 2.12	2.45 ± 2.47	1.62 ± 1.75	0.20^**b**^
TRAb (IU/L)	6.40 ± 4.98	3.66 ± 1.75	8.68 ± 5.65	**<0.001** ^**b**^
TGAb(IU/ml)	20.74 ± 14.72	20.46 ± 12.89	20.97 ± 16.36	0.91^b^
TPOAb(IU/ml)	42.08 ± 58.85	53.59 ± 80.04	32.48 ± 31.15	0.24^b^
NLR	2.07 ± 0.89	1.64 ± 0.45	2.42 ± 1.01	**<0.01** ^**b**^

Continuous covariates are shown as mean ± SD, and categorical covariates are shown as the number of events observed. The CAS was expressed as a median with range. Statistically significant *p*-values are in bold. *p*-values: ^a^ chi-squared test; ^b^ an independent samples t-test; ^c^ a Mann–Whitney U-test.

GD, Graves’ disease; GO, Graves’ orbitopathy; CAS, clinical activity score; BCVA, best-corrected visual acuity; logMAR, logarithm of angle resolution; MD, mean deviation; PSD, pattern standard deviation; VFI, visual field index; T3, triiodothyronine; T4, thyroxine; TSH, thyroid-stimulating hormone; TRAb, thyroid-stimulating hormone receptor antibody; TGAb, anti-thyroglobulin antibodies; TPOAb, anti-thyroid peroxidase autoantibody; NLR, neutrophil-to-lymphocyte ratio.

The BCVA across all eyes was 0.57 ± 0.56 logMAR, with a statistically significant difference between the groups (responsive outcome group: 0.27 ± 0.26 logMAR vs. unresponsive outcome group: 0.82 ± 0.62 logMAR, *p* < 0.001, Table 1). At diagnosis, the majority of patients exhibited color vision defects (77%) and orbital apex crowding on CT scan (93%), but no significant differences were observed between the two groups. Optic disc swelling or a definite relative afferent pupillary defect was observed in 17 eyes (39%), with a statistically significant difference between responsive and unresponsive groups (*p* < 0.001). Proptosis differed significantly between the two groups (*p* = 0.04), with an overall mean of 21.47 ± 3.62 mm. The visual field defects were significantly worse in the non-responsive group (MD: −5.56 ± 6.67 vs. -15.02 ± 10.14; *p* < 0.001, PSD: 4.07 ± 2.47 vs. 6.23 ± 3.45; *p* = 0.03, VFI: 84.45 ± 22.34 vs. 56.29 ± 35.35; *p* < 0.01). Laboratory analyses revealed that TRAb and NLR were significantly higher in the responsive group (3.66 ± 1.75 IU/L vs. 8.68 ± 5.65 IU/L; *p* < 0.01, 1.64 ± 0.45 vs. 2.42 ± 1.01; *p* < 0.01, respectively).

### 3.2 Primary outcome analysis

**Figure 2:**
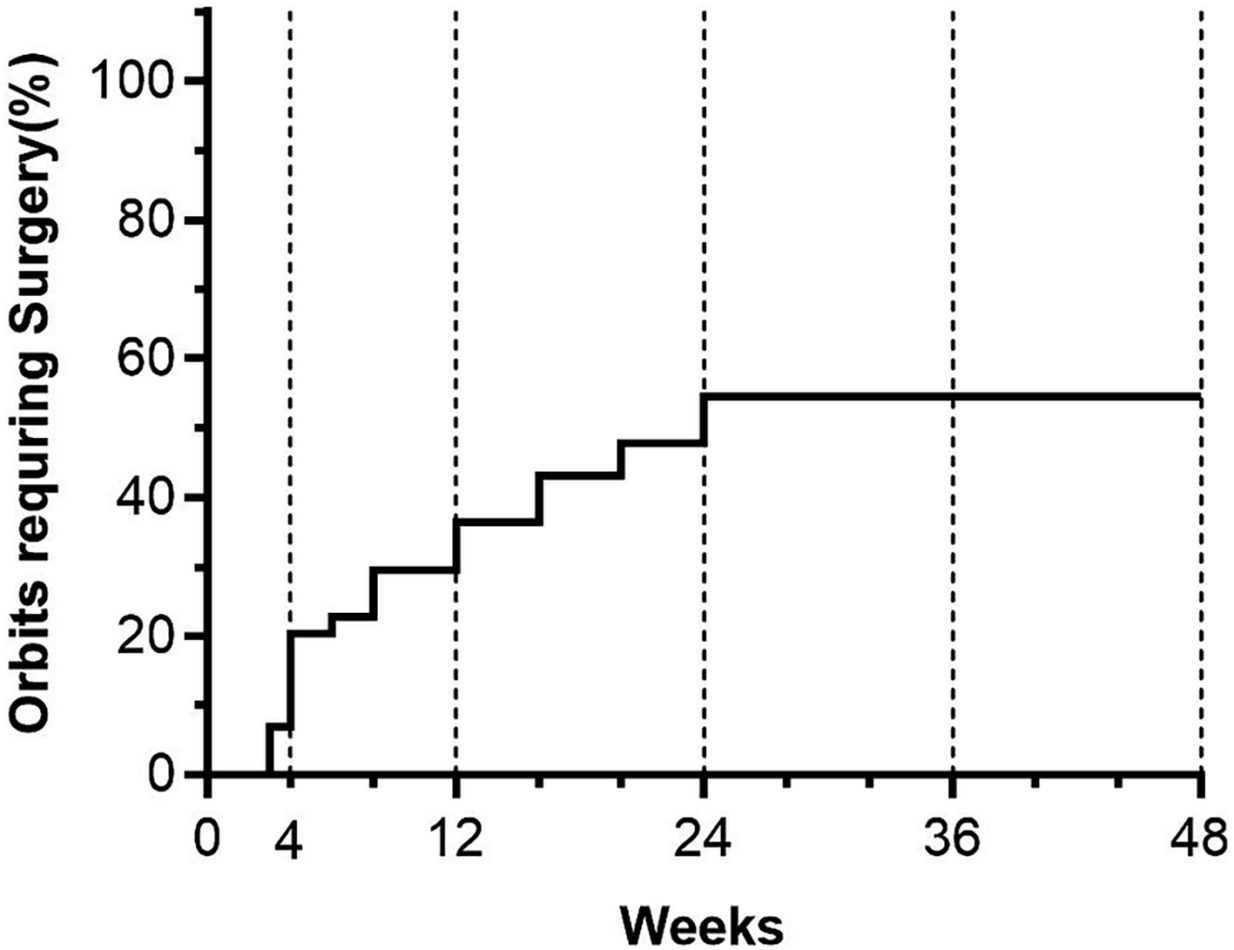
Percentage of the 44 eyes treated with high-dose intravenous methylprednisolone that required surgery at different times of follow-up. On the *x*-axis, the time (weeks) after medical decompression is shown. The dotted vertical reference lines mark the post-treatment follow-up times.

At the 6-month follow-up, 20 of the 44 eyes (45.5%) showed complete visual function recovery without subsequent recurrence of DON and did not require orbital decompression surgery. Among the remaining eyes, orbital decompression surgery was performed between 3 and 24 weeks (Figure 2; median = 8 weeks; 95% CI [6–16 weeks]) due to an inadequate response to steroid therapy or DON recurrence. In eyes that underwent orbital decompression surgery, no recurrence of DON was observed during the 6-month evaluation period.

**Figure 3:**
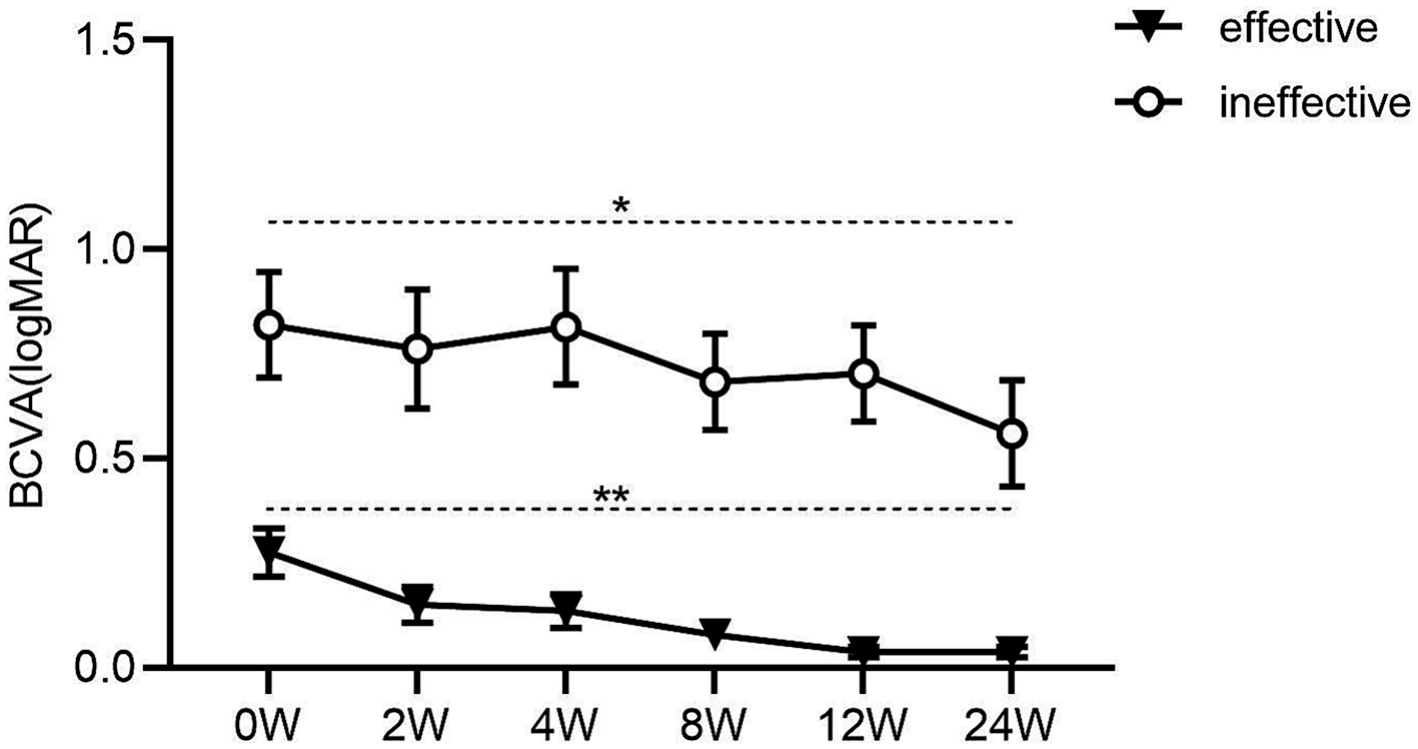
Changes in best-corrected visual acuity (BCVA, logMAR) after medical decompression up to 24 weeks of follow-up. Visual outcomes of eyes responding to intravenous methylprednisolone are shown with solid triangles, while those of eyes not responding are shown with empty circles. Values are shown as mean±standard error (SE). (*p < 0.05, **p < 0.01, ns = not statistically significant).

At the 6-month evaluation, visual function had improved significantly in both groups, independent of final outcomes (Figure 3, *p* < 0.01 in the responsive group vs. *p* = 0.04 in the unresponsive group). This improvement observed in the unresponsive group is attributable to the beneficial effect of orbital decompression surgery on optic nerve compression, as some patients in the unresponsive group had not yet undergone surgery in the preceding period. At the 2-month follow-up (mean BCVA = 0.08 logMAR) and thereafter, visual parameters remained stable and normal in all responsive eyes. Throughout the study, BCVA remained significantly lower in the unresponsive group than in the responsive group (*p* < 0.001).

### 3.3 Model construction of the HD-IVMP response in DON

**Figure 4:**
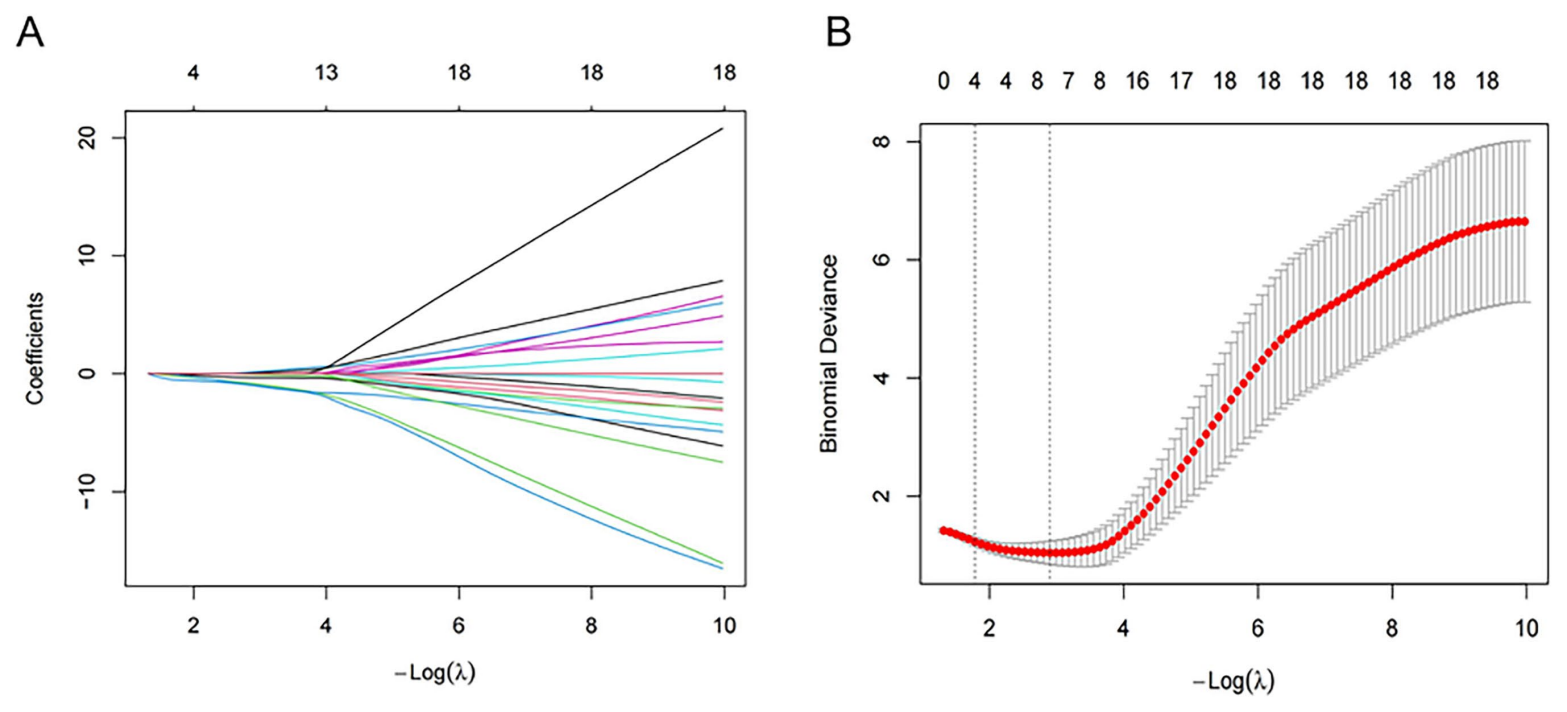
Feature selection using the LASSO binary logistic regression model. **(A)** The LASSO coefficient profiles of the 24 features. A coefficient profile plot was produced against the log (lambda) sequence. **(B)** Parameter selection in the LASSO model used tenfold cross-validation. The vertical dashed line at the optimal value was drawn via the minimum criteria (min. criteria) and one standard error of the minimum criteria (1se criteria). Partial likelihood deviation (binomial deviation) curves and logarithmic (lambda) curves were plotted. The optimal lambda produced four non-zero coefficients.

Based on LASSO logistic regression analysis (Figure 4), the 1-standard error (1-SE) criterion selected four variables with non-zero coefficients from 24 variables listed in Table 1: BCVA, TRAb, NLR, and optic disc swelling. These factors were subsequently incorporated into the multivariable stepwise logistic regression analysis, as shown in Table 2. The analysis revealed that, in the cohort of DON patients, baseline BCVA, TRAb, and NLR, rather than optic disc swelling, were significantly correlated with HD-IVMP treatment response (OR = 1.43, 95% CI: 1.05–1.95, *p* = 0.04; OR = 1.62, 95% CI: 1.02–2.61, *p* = 0.04; OR = 1.30, 95% CI: 1.04–1.63, *p* = 0.02, respectively).

**Table 2:** Multivariate stepwise logistic regression analysis of preoperative risk factors.

Variables	OR	95% Cl	*p-*value
BCVA (logMAR)	1.43	1.05–1.95	**0.04**
Thyroid laboratory results (normal range)			
TRAb (IU/L)	1.62	1.01–2.61	**0.04**
NLR	1.30	1.04–1.63	**0.02**

Odds ratios were calculated with the use of logistic regression analysis. Statistically significant *p*-values are in bold.

**Figure 5:**
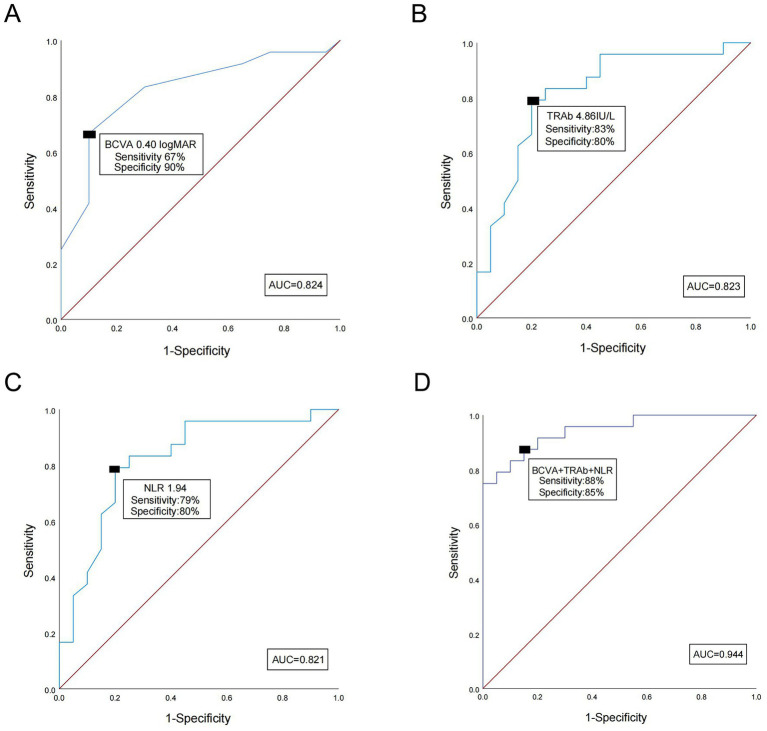
Receiver operating characteristic (ROC) curve analysis. **(A)** The ROC curves for best-corrected visual acuity (BCVA). The cutoff value of 0.40 logMAR showed a sensitivity of 67% and a specificity of 90% (AUC, 0.824; 95% CI, 0.70–0.95). **(B)** The ROC curves for thyroid-stimulating hormone receptor antibody (TRAb). The cutoff value of 4.86 IU/L represented a sensitivity of 83% and a specificity of 80% (area under the curve [AUC], 0.823; 95% CI, 0.70–0.95). **(C)** The ROC curves for the neutrophil-to-lymphocyte ratio (NLR). The cutoff value of 1.94 showed a sensitivity of 79% and a specificity of 80% (AUC, 0.821; 95% CI, 0.69–0.95). **(D)** The ROC curves for TRAb, NLR, and BCVA. The cutoff value showed a sensitivity of 88% and a specificity of 85% (AUC, 0.944; 95% CI, 0.88–1.00).

The predictive performance of individual and combined factors for HD-IVMP response in DON patients was evaluated using ROC curve analysis (Figure 5). Analyses revealed that the area under the curve (AUC) values for BCVA, TRAb, NLR, and their combined predictors were 0.824, 0.823, 0.821, and 0.944, respectively. The optimal cutoff values for each indicator were determined using the Youden index: 0.40 logMAR for BCVA, as shown in Figure 5A, with a sensitivity of 67% and a specificity of 90% (95% CI: 0.70–0.95); 4.86 IU/L for TRAb, as shown in Figure 5B, with a sensitivity of 83% and a specificity of 80% (95% CI: 0.70–0.95); 1.94 for NLR, as shown in Figure 5C, with a sensitivity of 79% and a specificity of 80% (95% CI: 0.69–0.95); and 0.48 for the combining predictor, as shown in Figure 5D, with a sensitivity of 88% and a specificity of 85% (95% CI: 0.88–1.00). These results indicate that the combined predictive model demonstrates superior sensitivity compared to any single predictor in assessing the HD-IVMP response in DON patients, as well as better specificity than BCVA.

### 3.4 Performance evaluation of the predictive models

**Figure 6:**
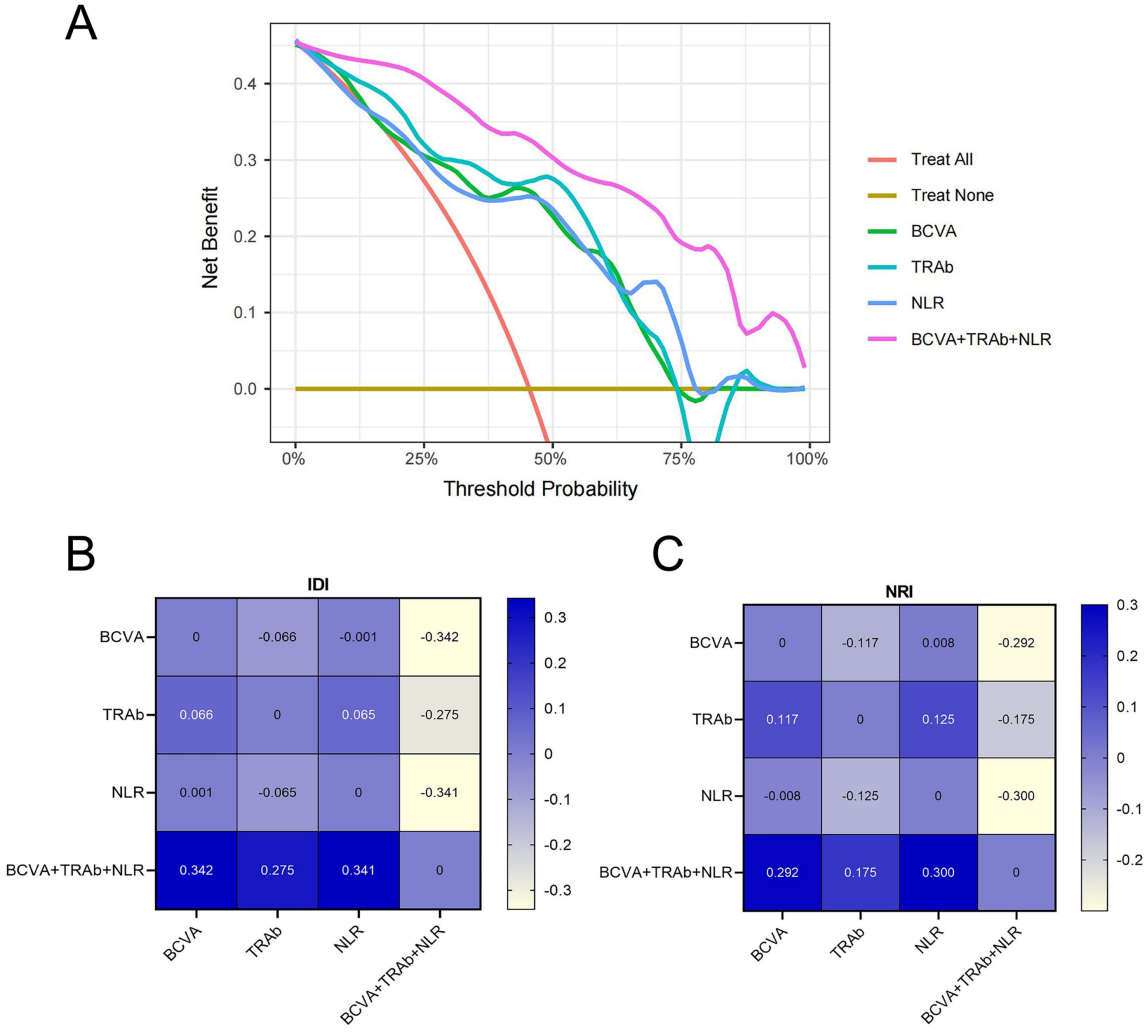
Clinical evaluation of models in the internal validation cohort. **(A)** The decision curve analysis of the prediction model (BCVA, TRAb, NLR, and combination model—BCVA+TRAb+NLR). The combination model showed the best benefit. **(B)** Integrated discrimination improvement of the models. **(C)** Net reclassification index of the models. The difference between the combined-factor model and the single-factor model represents the degree of improvement between the models; greater than zero indicates the effectiveness of the combination model. NRI, net reclassification index; DCA, decision curve analysis; IDI, integrated discrimination improvement.

In this study, we also evaluated both the single model and the combined model using calibration curves and DCA. The results revealed that, compared to scenarios in which no prediction model was used (i.e., treat-all or treat-none schemes), implementing interventions based on the combined model yielded greater net benefit at specific predictive probability thresholds, outperforming single-factor models (as shown in Figure 6A). Furthermore, model performance progressively improved with the inclusion of additional features, which was reflected in the combined model demonstrating significant gains over single-factor models in both the NRI (Figure 6B) and the IDI (Figure 6C). These findings indicate that the combined model offers superior clinical utility for predicting response to HD-IVMP.

**Figure 7:**
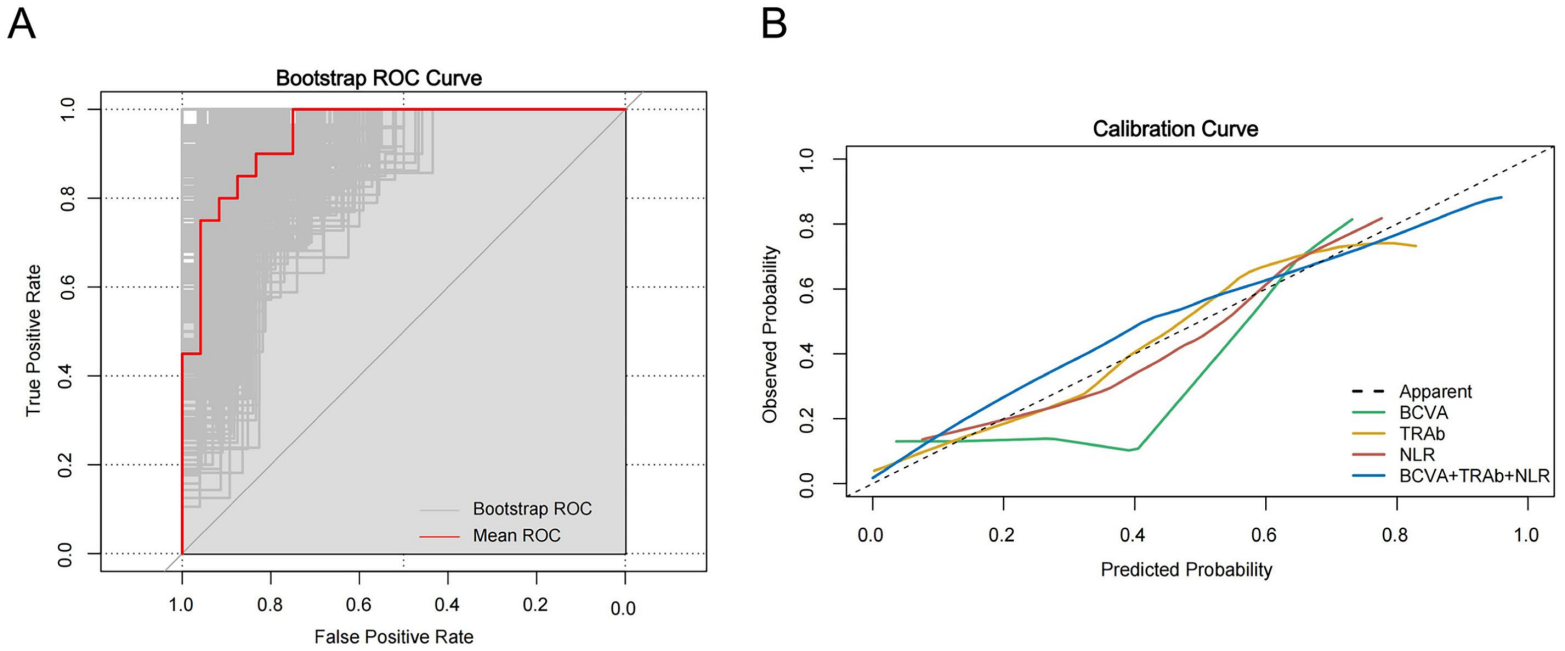
1,000 rounds of internal validation using bootstrap resampling of the model. **(A)** ROC analysis showing the combination model as a predictor of mean ROC performance in the red line and bootstrap ROC in the gray line. **(B)** Calibration plot of actual probability versus predicted probability of response to HD-IVMP according to the model. The closer the curve is to the center line, the more stable the model is.

The internal validation cohort was performed using 1,000-repetition bootstrap resampling of the model (Figure 7). The mean AUC was 0.944 (95% CI: 0.88–1.00), as shown in Figure 7A, and the accuracy was calculated to be 62%. The results demonstrate that the bootstrap estimates are largely consistent with the model’s original performance indicators, reflecting that the combination model remains robust even under a limited sample size. Although a certain discrepancy exists between the mean AUC and the bootstrap AUC, this phenomenon is likely attributable to sampling variability arising from the relatively small sample size. Furthermore, the calibration curve is presented in Figure 7B, indicating good agreement between the predicted and observed outcomes of the combination model in our cohort.

## 4 Discussion

This study presents the first dedicated analysis of risk factors predicting a poor response to HD-IVMP in DON patients who ultimately required surgical intervention. HD-IVMP serves as the first-line treatment for DON, aiming to alleviate optic nerve compression through drug-induced decompression. Identifying risk factors for a poor response to HD-IVMP treatment is crucial for guiding the optimal timing of surgical decompression. The study reveals a significant correlation between BCVA, TRAb, and NLR levels and a poor response to HD-IVMP. These results provide essential reference points for clinicians in formulating medical and surgical decompression strategies.

Although recent research has indicated novel therapeutic agents such as teprotumumab (23) and tocilizumab (24) as promising treatments for steroid-resistant DON, with rapid symptom improvement and long-lasting recovery, their current high cost limits widespread clinical adoption. In addition, our research enables the early identification of patients unlikely to respond to hormone therapy based on predictive factors. Specifically, for patients at risk of hormone resistance, early combination therapy with monoclonal inhibitors and IVMP can be initiated, thereby potentially reducing the need for decompressive surgery.

Until recently, several studies have described the efficacy of the recommended DON therapy (7, 25–27), including improvement of visual acuity, color vision, and visual field. Currently published literature has adopted a variety of DON improvement indicators, but no universally accepted criteria for defining DON resolution have been established. Our study defined eyes that did not require surgical decompression as having a good response to HD-IVMP treatment in DON patients; the observed response rate of 45.5% (20 of 44 eyes) alone aligns with previously reported rates. Mourits et al. (28) conducted a retrospective study on DON patients treated with HD-IVMP, showing a long-term remission rate of 39%. Post-treatment visual acuity assessment served as the key indicator for treatment success (BCVA> 0.5). Hart et al. (29) defined a two-line improvement in BCVA on the Snellen chart as a good response to treatment in patients with DON and indicated an overall long-term response rate of approximately 60%. Nicola et al. (7) reported complete visual recovery without surgery in 42.5% of DON eyes. The above studies collectively demonstrate that a significant proportion of DON patients exhibit poor responsiveness or even complete resistance to hormonal therapy. Given the potential for irreversible visual loss from prolonged optic nerve compression, early identification of such patients is crucial for guiding treatment strategies and avoiding delays in effective intervention.

Consistent with prior studies (4, 16, 30), we identified poor baseline BCVA as a significant risk factor for non-response to HD-IVMP treatment of patients with DON. Tagami et al. (4) reported that patients with baseline BCVA worse than 0.7 logMAR were more likely to require orbital surgical intervention due to failure of medical decompression. Garip et al. (31) observed that patients with a baseline BCVA of 0.3 logMAR achieved better treatment outcomes than those whose BCVA was worse than 0.6 logMAR. Parinee et al. (11) also observed that poor BCVA increased the risk of non-response to HD-IVMP treatment. Correspondingly, the results of this study showed that, when baseline BCVA was worse than 0.40 logMAR (the cutoff value), the odds ratio of unresponsive outcomes increased by 1.43 times.

We discovered that elevated TRAb levels are also an independent factor contributing to HD-IVMP non-response. GO is an autoimmune thyroid disorder fundamentally driven by TRAb-mediated activation of orbital fibroblasts. Studies have shown that TRAb levels are significantly elevated in the orbital tissues of patients with GO (32) and are closely associated with the clinical disease activity and severity (33). This finding can be explained by the fact that elevated TRAb levels may prolong the activation time of orbital fibroblast receptors, leading to increased orbital tissue volume and exacerbated compression of the optic nerve (34). Therefore, TRAb levels have been established as an independent risk factor and biomarker for GO in several studies (35, 36). This study further confirms that baseline TRAb levels serve as a significant predictor of the need for surgical intervention. When baseline TRAb levels exceed the cutoff value of 4.86 IU/L, the model predicts the need for surgical intervention with a sensitivity of 83% and a specificity of 80%. The value was significantly lower than the established cutoff (8.305 IU/L) for predicting hormone therapy efficacy in GO (10), suggesting that DON may require a lower inflammatory threshold to achieve a favorable response to IVMP. Interestingly, this finding aligns with prior research on tocilizumab therapy for preventing surgery in hormone-resistant DON, in which a baseline TRAb cutoff of ≤5.07 IU/L was associated with avoidance of surgery (24).

Furthermore, we detected NLR as a novel and readily accessible inflammatory marker associated with a poor response to HD-IVMP treatment. If the NLR exceeds 1.94, treatment with HD-IVMP may not yield favorable outcomes, necessitating subsequent decompressive surgery. An elevated NLR reflects a systemic inflammatory state; neutrophilia accompanied by lymphopenia indicates tissue injury, stress response, and inflammatory reaction and has been associated with poorer prognosis in various inflammatory diseases (17–19) and thyroid-related diseases (20–22). Previous studies have identified an association between higher NLR and increased GO activity (20, 36). In accordance with our findings, Nausicaa et al. have found that bronchoalveolar lavage fluid with high NLR was associated with a poor clinical outcome, influencing therapeutic response to high-dose corticosteroid therapy (37). As increased systemic inflammatory burden may lead to more aggressive or persistent disease, immunomodulation with glucocorticoids alone proves less effective.

Our results suggest that establishing appropriate cutoff values of these factors can assist surgeons in determining the optimal timing for orbital surgery. BCVA, TRAb, and NLR hold great clinical value due to their ease of acquisition, time efficiency, and cost-effectiveness. Early orbital decompression surgery is recommended for patients with elevated NLR and TRAb and poor BCVA due to the potential for a poor response to HD-IVMP after a 2-week course of medical decompression. However, our research indicated that any single-factor predictor has limited predictive value, exhibiting either low sensitivity (TRAb with a sensitivity of 83%, NLR with a sensitivity of 79%, BCVA with a sensitivity of 67%) or low specificity (TRAb with a specificity of 75%).

Based on this finding, we developed and validated a predictive model integrating the three factors. When the NLR and TRAb were elevated above 1.94 and 4.86 IU/L, or when BCVA was worse than 0.40 logMAR, respectively, the likelihood of non-response to HD-IVMP in DON ranged from 70 to 80%. While the predictive combined model (AUC = 0.944) demonstrated markedly improved sensitivity (88%) compared to any single parameter, as well as higher specificity (85%) than TRAb and NLR, it indicates significant value in predicting the optimal timing for surgical intervention while substantially reducing false-negative and false-positive rates.

Additionally, DCA of the combination model demonstrated superior net benefit across a wide, clinically relevant threshold probability range than single factors. Furthermore, the IDI and NRI demonstrated significant improvement in benefit. The internal validation confirmed the model’s robust clinical utility via 1,000-round bootstrap resampling. This indicates that the combination model may help to avoid unnecessary hormone therapy and enable timely surgery for patients with a poor response to HD-IVMP, thereby preventing irreversible vision loss. Although the precise relationship between these factors and response to HD-IVMP remains unclear, further research is needed to investigate the underlying mechanisms.

Furthermore, this study has certain limitations. The study’s retrospective design and reliance on data from a single center may introduce selection bias. Additionally, the upper limit of the confidence interval for the AUC of the ROC curve reached 1.00, indicating a consequence of the limited sample size. This ceiling effect suggests a risk of overestimation due to overfitting. To address this, external validation using independent, multi-center prospective cohorts is necessary to further confirm the robustness of our predictive model and the proposed cutoff values. Furthermore, the 6-month follow-up period, while sufficient for assessing initial treatment response, may be inadequate to capture long-term DON recurrence rates. Future prospective studies with extended follow-up are required to evaluate the model’s performance in predicting sustained outcomes.

In conclusion, this study demonstrates that poor BCVA and elevated TRAb and NLR are significant risk factors for a poor response to HD-IVMP treatment in DON patients. While these factors may not fully account for all non-responsive cases, our findings provide important reference criteria for identifying high-risk patients who are more likely to experience poor outcomes following medical decompression therapy. These factors, being readily available and cost-effective, can assist clinicians in determining the optimal timing for orbital decompression surgery, thereby offering valuable guidance for the treatment of patients with DON.

## Data Availability

The raw data supporting the conclusions of this article will be made available by the authors, without undue reservation.
